# Health, Housing, and Justice: Two‐Year Implementation Evaluation of a Health System's Multi‐State Medical‐Legal Partnership to Address Housing Instability

**DOI:** 10.1111/1475-6773.14620

**Published:** 2025-03-26

**Authors:** Natasha Arora, Sarah Terry, Holly R. Stevens, Vanessa W. Davis, Gerson Sorto, Bethany Hamilton, Marisa Conner, Alejandra Cabrera, Shane R. Mueller, Maria Casoni, Hiba Elkhatib, Ellen Lawton, Elizabeth Tobin‐Tyler

**Affiliations:** ^1^ Center for Community Health and Evaluation Seattle Washington USA; ^2^ Legal Services Corporation Washington District of Columbia USA; ^3^ Kaiser Permanente Oakland California USA; ^4^ Neighborhood Legal Services of Los Angeles County Glendale California USA; ^5^ National Center for Medical‐Legal Partnership, Department of Health Policy and Management Milken Institute School of Public Health, the George Washington University Washington District of Columbia USA; ^6^ HealthBegins Pasadena California USA; ^7^ Kaiser Permanente Colorado Institute for Health Research Denver Colorado USA; ^8^ George Washington University Washington District of Columbia USA; ^9^ Department of Health Services, Policy and Practice Brown University School of Public Health Providence Rhode Island USA

**Keywords:** eviction prevention, healthcare training, housing, legal aid, medical‐legal partnership, social determinants of health, social health

## Abstract

**Objective:**

To assess reach and identify facilitators of and barriers to the implementation of housing‐focused medical‐legal partnerships (MLPs) within a large healthcare system.

**Study Setting and Design:**

In 2021, Kaiser Permanente (KP) launched the Health, Housing, and Justice (HHJ) Initiative to embed MLPs within five medical centers across four states. KP invested in the capacity of five publicly funded legal aid providers to collaborate with healthcare teams and focus on housing stability. This paper summarizes findings from a mixed‐methods implementation evaluation conducted from 2021 to 2023 on staff and system capacity, operational facilitators and barriers, and lessons learned.

**Data Sources and Analytic Sample:**

Data sources included key informant interviews with healthcare and legal staff, surveys of social workers and care navigators, and administrative data on 857 legal referrals made by medical staff in 2022–2023 for housing‐related legal support.

**Principal Findings:**

Implementation characteristics and the rate of referrals varied across each of the six sites engaged in the multisite MLP. Attorneys reported that the MLP enabled access to legal resources for clients who typically would not have access. Most cases (82%) were addressed with fewer than 5 h of attorney time. Key implementation facilitators included clinical champions in the partnering medical team, staff training with a focus on knowledge of housing‐related legal issues and MLP referral criteria, and existing social screening processes. Key implementation barriers were associated with information sharing, orienting legal partners to a complex medical system, and mismatches in service delivery areas between KP and the legal aid organizations.

**Conclusions:**

Embedding MLPs upstream in healthcare systems can enable access to legal resources for underserved clients. Attention to key implementation factors can support the spread of MLPs within other large healthcare systems.

**Summary**

- What is known on this topic
  ○ Access to legal representation helps tenants at risk of eviction or housing instability maintain safe, stable housing.
  ○ Medical‐legal partnerships, which partner legal and healthcare teams to identify and remedy health‐harming legal needs for low‐income patients, are an effective intervention to address social needs, including housing.
  ○ Healthcare systems increasingly invest in interventions that bolster patients' housing stability because of the adverse impact of housing instability on health, health equity, continuity of care, and health care costs.
- What this study adds
  ○ Understanding of MLP implementation across multiple clinical sites to inform MLP scaling activities in a range of settings.
  ○ Understanding of key implementation facilitators, including staff training and robust social needs screening processes.
  ○ A blueprint for integrating MLPs into healthcare systems, highlighting the role of tailored processes, infrastructure, and cross‐sector collaboration in addressing housing‐related legal needs.

## 1 Introduction

Healthcare system‐based interventions that address social needs have proliferated in the past decade [1]. Long before the current trend, medical‐legal partnerships (MLPs), which partner legal and healthcare teams to identify and remedy health‐harming legal needs of low‐income and underserved populations, took root in the early 1990s. MLPs are active in 175 hospitals and health systems, up to 150 community health centers, 50 Veterans Affairs medical centers, and 83 other clinical settings in 49 states and the District of Columbia; 170 legal aid agencies participate in MLPs [2, 3].

According to the 2022 Justice Gap Report, an estimated 74% of low‐income households in the United States experienced at least one civil legal problem [4]. Frequent civil legal needs include securing and protecting access to housing, health care, and income [4].

Complexity of legal needs varies substantially. Some people at risk of housing instability may require small “doses” of legal intervention to maintain their housing, such as help securing temporary rental or a letter to a landlord asserting the right to accommodations for a disability. Other housing and related legal needs are more complex and require intensive legal support, such as representation in court.

The establishment of a legal “continuum of care” that integrates legal resources within the healthcare setting improves opportunities to prevent legal issues. This approach aligns with the shared goals of the health, public health, and legal sectors, to foster health justice and improve well‐being for individuals and communities [5, 6].

The MLP intervention embeds access to civil legal aid services within healthcare organizations to address patients' immediate social needs. While MLPs address a range of social and structural drivers of health, including access to income supports and government benefits, employment issues, and domestic violence, housing instability is the most cited barrier to health confronted by MLP teams [7]. Evictions have reached a crisis point in many states and cities across the country [8, 9]. Meaningful access to legal advice and representation is demonstrated to help tenants maintain safe, stable housing and avoid homelessness [9].

In 2021, Kaiser Permanente (KP) launched the Health, Housing, and Justice Initiative (HHJ Initiative) to embed MLPs in the KP system. KP made a $3.5 M investment to partner with five publicly funded legal aid teams in four states, serving six regions: Legal Services of Northern California, Neighborhood Legal Services of Los Angeles County, Maryland Legal Aid, Legal Aid Services of Oregon, and Colorado Legal Services. Each publicly funded legal aid provider received an individual, flexible grant to increase legal aid staffing.

Capacity‐building support was provided by KP's national Community Health team and a national training and technical assistance (T/TA) team funded by KP and co‐led by the National Center for Medical Legal Partnership (NCMLP) and HealthBegins.

The investment by KP builds upon two trends: (1) healthcare system investment in MLP as a social determinant of health intervention that addresses individual needs as well as structural and policy factors and (2) healthcare system investment in interventions that bolster patients' housing stability because of the adverse impact of eviction on health, health equity, continuity of care, and health care costs [5, 10]. Despite the proliferation of interventions addressing health‐related social needs and evidence of the effectiveness of MLPs, investment by a large healthcare system in a national MLP pilot is unprecedented [11].

KP conducted an implementation evaluation of the multi‐site HHJ initiative. This mixed‐methods implementation evaluation sought to (1) understand the extent to which the MLP reached and served patients within the KP system and (2) identify implementation facilitators and barriers. An outcomes evaluation will be conducted separately.

## 2 Methods

The evaluation team conducted a mixed‐methods implementation evaluation, using data from key informant interviews, surveys of case managers, administrative data on referrals and legal cases, and observation of program activities and review of documents.

### 2.1 Study Setting

MLPs were embedded within six sites located in four states within the KP system beginning in November 2021. Each “site” refers to a regional team of social workers, care navigators, or nurse case managers (referred to as case managers moving forward) that received social needs referrals from physicians or conducted initial social needs intake assessments with new KP Medicaid members. Case managers operated differently and served different patient populations within each region; some received social needs referrals from providers located in various departments and medical centers, while others served caseloads of particular high‐needs patients. Most contacts between case managers and patients were virtual, conducted by telephone or video telehealth.

At each site, attorneys trained social work and case management teams on housing‐related legal topics, including housing insecurity and identifying patients with housing‐specific legal issues, using a combination of office hours and informal consultations referred to as “curbside consultations.” These brief, healthcare‐initiated interactions with the legal aid team helped assess whether a patient's situation warranted a formal referral [12]. Attorneys provided legal services to patients, ranging from brief consultations to representation in court. In February 2023, standardized training for case managers was enhanced with the introduction of uniform training materials on housing‐related legal issues, developed by the T/TA team.

### 2.2 Data Sources and Analysis Approach

#### 2.2.1 Administrative Data

Descriptive statistics of administrative data from two sources, by site, were generated using Microsoft Excel software. The time period for administrative data collection was December 2021–November 2023.

1. Referrals to the legal partner from KP's Unite Us‐based social needs referral platform. Referral data included client demographic data, referral date, referral status, referring case manager, and a free‐text case description field. Referral data, accessible to both medical and legal partners, included identifying information to support case management collaboration. However, data were de‐identified for evaluation purposes.
2. Information from legal aid data/case management platforms. All legal aid organizations used LegalServer or JusticeServer to document their interactions and case outcomes with clients and shared the following data with the evaluation team: client demographic data, problem codes, legal case status, hours spent on legal cases, and legal case outcome category. Case data included identifiers for the legal team to facilitate their work but were de‐identified for evaluation purposes. These data were not accessible to the medical partner.

Additionally, at a single timepoint upon joining the initiative, sites were asked to report (1) the number of unique patients per year seen by participating departments, (2) the roles of case managers involved within the MLP (e.g., social workers, nurse case managers, community health workers, etc.), and (3) the number of case managers in participating MLP departments.

Administrative data were used to calculate (1) referral rate (number of referrals made per 10,000 unique patients), (2) percentage of case managers who made at least one referral, and (3) percentage of case managers who requested at least one curbside consult.

#### 2.2.2 Key Informant Interviews

Between September 2021 and December 2023, the evaluation team conducted 89 semi‐structured interviews, ranging in length from 15 to 55 min with local KP leadership and legal aid providers at each active MLP site, case managers who had made referrals to the MLP, and members of the T/TA team who supported the initiative. A total of 69 unique individuals participated in at least one interview; some were interviewed multiple times. Interviewees were asked about implementation progress at their site, as well as facilitators and barriers to implementation.

Each interview was recorded, transcribed, and coded by one of three coders. Thematic analysis was used to identify themes across interviewees and sites [13]. Both deductive and inductive methods were used. Deductive methods were applied by coding interviews using a list of categories developed based on an implementation guide published by NCMLP, including key implementation domains: partnership development, screening and referrals, bi‐directional training, information‐sharing, and delivery of legal services. Inductive methods were used to identify additional emerging themes. After themes were developed, they were reviewed at two time points by medical and legal team leads and by members of the initiative T/TA team to ensure validity of findings and allow for multiple initiative stakeholders to contribute to interpretation of qualitative data.

#### 2.2.3 Observation and Document Review

To gain relevant context for key informant interviews, the evaluation team observed and took notes at planning meetings, learning events, and occasional office hours held by legal aid providers at the four initial MLP pilot sites. They reviewed tools, resources, meeting notes, and agendas for MLP‐related meetings. Thematic analysis, as described in the key informant interview section above, was applied to notes from observations and document review.

#### 2.2.4 Triangulation Across Sources

At the conclusion of the evaluation, themes from individual data sources were triangulated across sources to identify consistent, cross‐cutting themes across the six MLP sites and site‐level factors that contributed to successful MLP implementation. Quantitative data sources were used to provide implementation context for each site (e.g., number of referrals made, number of staff who made referrals, and number of legal services provided); qualitative data sources were used to derive key themes related to implementation facilitators and barriers.

#### 2.2.5 IRB Review

This study was exempted from review by the Kaiser Permanente Institutional Review Board because the study was for quality improvement purposes.

## 3 Results

### 3.1 Administrative Data

Over a period of 24 months (December 2021–November 2023), case managers made a total of 857 patient referrals to partnering legal aid attorneys. Across all sites, a total of 132 case managers made referrals to the MLP, and 75 case managers requested curbside consults from partnering attorneys. Table 1 displays site‐specific characteristics and measures of implementation progress, including the number of referrals made and the number of staff who made at least one referral or requested at least one curbside consult. Data from site C were incomplete, as they did not report the number of patients seen annually or the number of case managers. Departments participating in the MLP differed across the six sites. At some sites (sites A, B, and D), participating departments included regional teams that addressed social health referrals generated by medical providers across a relatively large geographic region encompassing multiple medical centers; at other sites, participating departments included case management teams that worked with medical providers at a specific medical center (site E) or supported intensive case management for a relatively small group of patients (sites A and C). Some sites included case managers working in multiple departments (sites A and F). The rate of referrals made per 10,000 patients could be calculated for five sites for which data was available. In total across five sites, 129 referrals were made per 10,000 patients; the site‐specific rates ranged from 17 referrals per 10,000 patients (site F) to 475 per 10,000 patients (site A). The percentage of departmental staff making referrals could be calculated for five sites for which data were available, and the percentage of staff requesting curbside consults could be calculated for four sites. Percentages of departmental staff making referrals were 56% across all sites and ranged from 5% to 72% at individual sites; percentages of staff making curbside consults were 32% across all sites and ranged from 14% to 67% at individual sites.

**TABLE 1:** Implementation characteristics of MLP sites.

Site	Department description (multiple departments separated by semicolon)	Roles of case managers	Annual number of patients seen by site	Month of first documented referral	Number of referrals made to legal services (from date of first referral to Nov 2023)	Referrals per 10 K patients (from date of first referral to Nov 2023)	# of case managers in departments	# of case managers who made at least one referral (from date of first referral to Nov 2023)	% of case managers who made at least one referral	# of case managers who requested at least one curbside consult (from date of first referral to Nov 2023)	% of case managers who requested at least one curbside consult
A	Integrated care department serving patients with intensive case management needs; regional care coordination teams	Nurse case manager, social workers, peer community health workers, and registered nurses	9195	Dec 2021	437	475	54	39	72%	36	67%
B	Regional care coordination team	Community health navigator and social workers	11,700	Oct 2022	70	60	46	22	48%	Not reported by site	
C	Case management department for a specific medical center	Social workers, community navigator, and registered nurses	Not reported by site	Nov 2022	95	Could not be calculated	Not reported by site	17	Could not be calculated	8	Could not be calculated
D	Regional care coordination team	Community specialists, registered nurses, and social workers	23,978	Mar 2023	90	38	53	24	45%	13	25%
E	Care coordination team associated with specific medical center	Medical social workers	6470	Jun 2023	140	216	60	29	48%	15	25%
F	Inpatient social work; regional care coordination team	Social workers and registered nurses	15,000	Nov 2022	25	17	22	1	5%	3	14%
Total			66,343		857	129	235	132	56%	75	32%

Data from legal aid organization databases were reviewed in November 2023. At that time, 396 cases had been opened and 330 (83% of opened cases) were closed, as displayed in Table 2. Cases from sites D and E were excluded, given that those sites only began accepting referrals in mid‐2023 and had few closed cases.

**TABLE 2:** Services provided in closed cases (*n* = 330).

Services provided in closed cases		
Time spent	Closed cases	% of cases
Less than 1 h	40	12%
1.00–4.99 h	232	70%
5–9.99 h	35	11%
10–19.99 h	16	5%
20–49.99 h	5	2%
50+ h	2	1%
Final level of service		
Limited service (includes administrative agency decision, counsel and advice, and limited action)	314	95%
Extended service (includes negotiated settlement without litigation, negotiated settlement with litigation, extensive services, uncontested court decision, and contested court decision)	16	5%

At that time, 82% of cases used fewer than 5 h of attorney time and 95% involved provision of Limited Services as defined by Legal Services Corporation reporting codes, which are typically counsel and advice cases (i.e., attorneys provide advice to clients but do not represent them in court or take other legal actions on their behalf) [14] (see Table 2). Legal outcomes among closed cases were reported using the outcome codes established by the California Legal Aid Reporting and Evaluation Handbook [15], as displayed in Table 3. A majority of cases were coded with the outcome “Accessed client's right to the justice system,” which is used when other outcomes in the coding system do not apply and includes situations in which attorneys provide consultation and advice but do not result in defined outcomes related to housing or other needs. Thirty‐two percent of closed cases resulted in housing‐related outcomes, including prevention of eviction, facilitation of transition to other housing before eviction, and improvements to habitability. A smaller number of cases resulted in other outcomes related to legal status or personal and family stability.

**TABLE 3:** Legal outcomes (*n* = 288) among closed cases (*n* = 330; cases can have multiple outcomes).

Legal outcomes	Number of legal outcomes	Percentage
Access to justice system	**161**.	**56%**
Facilitated access to legal representation or advice (e.g., assistance with legal forms, consultations)	161	56%
Housing and utilities	**91**	**32%**
Received advice or brief counseling on housing matters (e.g., tenant rights, habitability issues)	29	10%
Prevented eviction or preserved housing stability	15	5%
Negotiated a “soft landing” (e.g., facilitated transition to alternative housing)	10	3%
Obtained other housing‐related benefits (e.g., utility relief)	32	11%
Enforced rights to safe and habitable housing (e.g., legal action against landlords for unsafe conditions)	5	2%
Removed barriers that impact employment, benefits, housing, and self‐sufficiency	1	< 1%
Legal status	**2**	**< 1%**
Obtained advice or resolution on immigration matters (e.g., visa eligibility) or provided documents in accessible formats for legal processes	2	< 1%
Personal and family stability	**16**	**6%**
Secured or preserved disability‐related rights, benefits, or accommodations	14	5%
Received advice, information, or referral on family matters	2	< 1%

Overall, administrative records on MLP cases and their outcomes indicate that most (82%) cases were addressed using fewer than 5 h of attorney time. About one‐third of cases referred to the MLP and 10% of overall referrals, including 91 closed cases, resulted in legal actions that supported clients in maintaining housing, exercising rights as a renter, improving habitability of their home, or obtaining a “soft landing” (i.e., transition to alternative environment) when facing eviction proceedings.

### 3.2 Interviews

#### 3.2.1 Attorney Perception of Services Provided

Legal aid attorneys who were interviewed about their experiences with the MLP (*n* = 9) indicated that direct referrals made by KP patient‐facing staff connected them with clients who would not typically access legal aid, including patients who would not otherwise be aware of legal aid services, patients whose health conditions or disabilities limited their ability to access services, and patients who would not have qualified for services based on legal aid organizations' standard eligibility criteria or triage decisions. Attorneys noted that the clients referred through the MLP were often more vulnerable to experiencing housing instability than typical legal aid clients, because of compounding effects of limited financial resources and health conditions. Attorneys described working with clients whose disabilities have both contributed to their housing crises and prevented them from effectively navigating the systems that could help mitigate housing‐related crises. One attorney described the ability to serve people whose limited mobility would prevent them from getting to court as “a huge success.” Another attorney stated that the MLP has supported their organizational goals around fighting poverty because “we [legal aid organization] want to serve our entire community. We don't want to sit in a silo and only serve those who know about us.”

When asked about the role of brief consultations, legal aid attorneys stated that brief consultations allowed them to support clients to take preemptive action to preserve their housing (e.g., by understanding their legal rights, maintaining records that would be needed later applying for financial assistance, etc.) or to apply for public benefits for which they did not understand they were eligible.

Attorneys referenced collaboration with KP staff as a facilitator of successful outcomes for some clients. When healthcare staff collaborated with legal aid attorneys, healthcare staff could support clients to follow through with the attorney's instructions (e.g., obtaining documents, documenting interactions with a landlord, etc.) or connect clients with resources related to needs that surfaced during their interactions with attorneys. Attorneys found that it was particularly helpful to have a direct connection within the KP system to secure medical documentation that was needed in legal situations, such as reasonable accommodation letters from physicians.

#### 3.2.2 Facilitators and Barriers to Implementation

Qualitative interview and observation/document review data were used to identify facilitators and barriers to implementation. Qualitative data were also triangulated with administrative data to assess differences in performance across sites in terms of total number of referrals made, number of referrals made per 10 K patients, and number and percentage of case management staff making referrals or submitting curbside consults. These metrics provided a way to measure the extent to which case management staff were engaged in the initiative and equipped to identify patients who would benefit from MLP services.

#### 3.2.3 Facilitators

##### 3.2.3.1 Clinical Champions

Within sites A, B, and C, clinical champions were engaged within the site's MLP leadership team. Their role was to promote the MLP to peers, respond to questions about potential patient referrals, and encourage staff to access tools developed for the MLP, such as job aides and screening tools embedded within the electronic health record. In an environment with high caseloads, many responsibilities among case managers, and frequent roll‐outs of new workflows, the role of the clinical champion was instrumental to directing peers' attention to the MLP and explaining to them how it differed from other social needs referral resources (i.e., the MLP as a collaborative, closed‐loop referral process, instead of a one‐time unidirectional referral).

##### 3.2.3.2 Staff Training

Attorneys reported that patient‐facing staff training resulted in high quality referrals that were well‐aligned with the eligibility criteria of the MLP and provided adequate information for attorneys to determine next steps. Case managers reported that the ongoing nature of trainings kept them engaged with the initiative and allowed them to learn about eviction law and other housing‐related legal topics. Case managers noted that housing and legal topics, a frequent area of need for patients, are often complex, and that trainings supported them to both develop their understanding of housing‐related legal issues and formulate questions to legal partners that would allow them to better support their patients. As one case manager said, “[The MLP] makes me feel a little bit more knowledgeable because housing is just this beast… There's just a lot of information, and I don't know the specifics, but I know to ask these questions.”

##### 3.2.3.3 Social Health Screening

KP's existing social health screening protocols supported integration of the MLP into case managers' workflows. Social health screening protocols were previously integrated into the KP health system, and patient‐facing staff making MLP referrals were familiar with social health screening at most sites, including sites A, B, D, E, and F. At sites A, D, and E, patient‐facing staff were also familiar with the use of KP's Unite Us‐based social needs referral platform. An additional patient identification tool with a focus on MLP eligibility criteria was developed by the MLP team at site A and distributed to the remaining MLP sites. One social worker described how both existing familiarity with social health screening and the clear criteria for MLP referral eligibility supported her identification of patients to refer to the MLP: “Part of our role as social workers is to screen for needs… The criteria for the program made it very simple to [identify MLP referrals]. When we're hearing about people having issues connected to benefits, or having issues with the landlord… or who were already evicted but unable to secure housing because it was part of their record… The information about who would meet criteria was pretty clear to me.”

#### 3.2.4 Barriers

##### 3.2.4.1 Information‐Sharing

Challenges with information‐sharing were reported by KP patient‐facing staff, including (a) inability to see updates from attorneys due to the constraints of legal organizations using KP's Unite Us‐based social needs referral platform, (b) some legal teams' requirement of written consent to share information back to the medical team (others permitted sharing with verbal consent), and (c) difficulty obtaining written consent from patients/clients to share information when services were provided digitally. Given that some information‐sharing challenges were the result of different requirements and norms about information‐sharing between medical and legal organizations, information‐sharing challenges and their possible solutions were raised as a discussion topic during monthly cross‐site legal team meetings, which did not include the medical partners. Written consent challenges were addressed by adopting digital signature software, though patients with barriers to accessing technology could not always provide consent through e‐signatures.

##### 3.2.4.2 Mismatches in Service Delivery Areas

Sites B and F did not generate consistent referrals. As seen in Table 1, site F had the lowest referral rate of the five sites for which data were available; site B had a higher referral rate but did not begin generating referrals until October 2022. A significant challenge at these sites was a geographic mismatch between the areas served by KP case management teams and the areas served by partnering legal aid organizations. Geographic mismatches meant that case managers or social workers who had been trained in making MLP referrals rarely encountered patients who would be eligible for services. Other times, KP leaders and case managers suggested that the confusing nature of criteria (i.e., patients in some geographic areas were eligible, while patients in others were not) made it more difficult for case managers to recall who would be eligible, and therefore less likely to assess for referral eligibility.

##### 3.2.4.3 Orienting Legal Partners to a Complex Health System

Legal leads, medical leads, and national T/TA team members all noted that the cultural norms of healthcare delivery and KP's organizational structure were challenging to navigate. Lack of familiarity with the KP system and with the roles of individual health care workers and leaders made it difficult to determine whom to approach when they experienced challenges collaborating. Attorneys shared that they would benefit from a deeper understanding of the healthcare context and of the services available to patients/clients from the healthcare side of the partnership, particularly given that in‐person collaboration and site visits were not features of this MLP at most sites. As one medical site lead noted, “If [the legal aid team is] new to medical‐legal partnership, or haven't worked with health care before, there's just a tremendous learning process around how the culture of health care works, what the expectations are in terms of how people show up, the pace of care delivery, and the demands of the team. It's just very different.”

## 4 Discussion

This study describes the results of a mixed‐methods implementation evaluation of the KP MLP, which included partnerships between six KP sites and five legal aid organizations. A total of 857 referrals were made to the MLP, at a rate of 129 referrals per 10,000 unique patients seen by participating medical departments. Most cases (82%) were addressed with fewer than 5 h of attorney time. Attorneys described that the MLP allowed them to see clients that would not otherwise reach them, sometimes due to medical needs or disabilities, and that collaboration across medical and legal teams supported clients to follow attorneys' advice. Clinical champions in the partnering medical team, staff training with a focus on knowledge of housing‐related legal issues and MLP referral criteria, and existing social screening processes were all important to the success of the MLP. Challenges included information sharing, mismatches in service delivery areas, and orienting legal partners to a complex medical system.

Unlike the healthcare system, the legal aid sector generally relies on individuals to self‐diagnose, seeking legal assistance when and if they can do so; there is no analogous system of primary and preventive care for people with legal needs [16]. Early and efficient collaboration between health and legal professionals increases capacity for effective allocation of specialized, scarce legal resources by allowing the MLP team to assess legal complexity and deliver the appropriate level of legal intervention to address the screened legal need. Access to virtual office hours for training and triage sensitizes healthcare team members to detect legal issues early.

While there is substantial evidence demonstrating the effectiveness of the MLP intervention, an implementation evaluation of MLP in a multi‐site, integrated health system is unprecedented [17]. Existing implementation‐related MLP literature predominantly consists of single‐site studies that focus on health system or workforce improvements within a single clinical context [2, 18]. This study also differs from previously studied MLP interventions in that (1) the primary health system actors within this MLP's context were case managers (including social workers, nurse case managers, and community health workers), rather than physicians and nurses; and (2) the intervention was conducted in a primarily virtual context as a result of the COVID‐19 pandemic, with most interactions (between patients and health care workers, health care workers and attorneys, and attorneys and clients) occurring virtually.

In comparing these results with smaller MLP studies, similarities shed light on adaptable practices that would promote scale, such as further standardization of screening and training processes [19]. Considering the broader landscape of social health interventions, it might be beneficial to juxtapose MLP implementation with similar initiatives, e.g., interventions addressing food insecurity or transportation barriers.

Limitations of the study findings include the following: (1) administrative data used to gauge the reach and progression of MLP activities were incomplete, as described in the results section; (2) while the data primarily focus on assessing the overall progress of MLP and extracting lessons to bolster sustainability and future expansion, they lack outcome data measuring the intervention's direct impact on patients. This aspect is addressed through a separate, ongoing outcomes evaluation; and (3) findings may not be generalizable to other contexts or healthcare systems due to unique characteristics of the implementation setting.

KP's MLP launched with four initial sites and quickly expanded to include two additional sites, leveraging infrastructure and lessons learned from the pilot phase. The national T/TA team, in collaboration with participating legal aid organizations and informed by evaluation findings, developed a publicly available blueprint for MLP implementation. This blueprint, including training materials on housing‐related legal topics, as well as workflows, job aids, and guides on promising implementation practices, is accessible on the NCMLP website and can be tailored for use in diverse institutional contexts [20].

These resources hold promise for replicating MLPs in settings such as the Health Resources and Services Administration's Health Center Program and the VA system, which share operational similarities with KP. Additionally, other large health systems can draw on these findings to launch or enhance their own existing or burgeoning MLP initiatives.

Future research is needed to evaluate the outcomes and cost‐effectiveness of MLPs, particularly with larger healthcare systems. Existing evidence suggests MLPs effectively address housing‐related legal needs among Medicaid populations [21]. Managed care organizations, government agencies, and other stakeholders should explore the feasibility of broader adoption, including the development of payment models that align with other health‐related social needs interventions, such as food security and housing services [22]. These efforts could position MLPs as a critical component of person‐centered and equitable healthcare [23].

MLP models require healthcare system investment to effectively address social needs, underscoring the imperative of allocating resources for capacity building and infrastructure development within both the health and legal aid systems. Such investment has the potential to not only enhance patient outcomes but also yield long‐term benefits by reducing healthcare costs associated with unaddressed SDOH [24].

## 5 Conclusions

MLP models show promise to support individuals at risk of housing insecurity. This implementation evaluation of a multi‐site MLP intervention demonstrates that many clients' legal challenges can be addressed with low‐intensity legal support and emphasizes the importance of staff training, social needs screening, and information‐sharing. Findings may serve as a blueprint for the development and scaling of future MLP interventions.

## Conflicts of Interest

Natasha Arora, Sarah Terry, Vanessa Davis, Marisa Conner, and Shane Mueller are salaried employees of Kaiser Permanente.
